# Supplementary material for: Molecular identification of avian influenza virus subtypes H5N1 and H9N2 in birds from farms and live bird markets and in respiratory patients
Source: PeerJ. 2018 Sep 5;6:e5473. doi: 10.7717/peerj.5473 (PMC6129142; doi:10.7717/peerj.5473)
Supplement: Supplemental Information 1 [file peerj-06-5473-s002.pdf]

AIV KNOWLEDGE, PRACTICES AND ATTITUDES IN SHARKIA GOVERNERATES

Participant name: ……………………………………………….... **Date:** ……………………

Interviewer: …………………………………………………………………………………….

Address: ………………………………………………………………………………………

I. DEMOGRAPHIC CHARACTERISTICS

1. Gender:

- ☐ Male
- ☐ Female

2. Age:

- ☐ ≤ 19 years
- ☐ 20-49 years
- ☐ ≥ 50 years

3. Educational level:

- ☐ Illiterate
- ☐ Primary school
- ☐ Secondary school
- ☐ High school
- ☐ University
- ☐ Other, specify ………………….

4. Occupations:

- ☐ Student
- ☐ Employer
- ☐ Farmer
- ☐ Household
- ☐ Other, specify ……………………

II. KNOWLEDGE

5. Have you heard about AIV?

- ☐ Yes
- ☐ No

6. AIV is infectious to humans

- ☐ Yes
- ☐ NO
- ☐ Don't know

7. Information source about AIV

- ☐ TV
- ☐ Radio
- ☐ Friends
- ☐ News
- ☐ Health facilities
- ☐ None, specify ....

8. Knowledge about methods of AIV transmission

- ☐ Yes
- ☐ No

9. Knowledge about symptoms in humans

- ☐ Yes
- ☐ No

III. ATTITUDES AND PRACTICES

10. Do you have direct contact with poultry?

- ☐ Yes
- ☐ No

11. Do you use preventive measures during contact with poultry?

- ☐ Yes
- ☐ No

12. What will you do if you are sick with fever, sneeze, and cough?

- ☐ Purchasing cold medicine by myself
- ☐ Seeking medical service from hospital
- ☐ Other, specify ....

13. Did you administrate any influenzas vaccines?

- ☐ Yes
- ☐ No
