# Supplementary material for: Molecular identification of avian influenza virus subtypes H5N1 and H9N2 in birds from farms and live bird markets and in respiratory patients
Source: PeerJ. 2018 Sep 5;6:e5473. doi: 10.7717/peerj.5473 (PMC6129142; doi:10.7717/peerj.5473)

Supplementary Figure S1. Amplification curve of human samples for the identification of avian influenza viruses H5N1

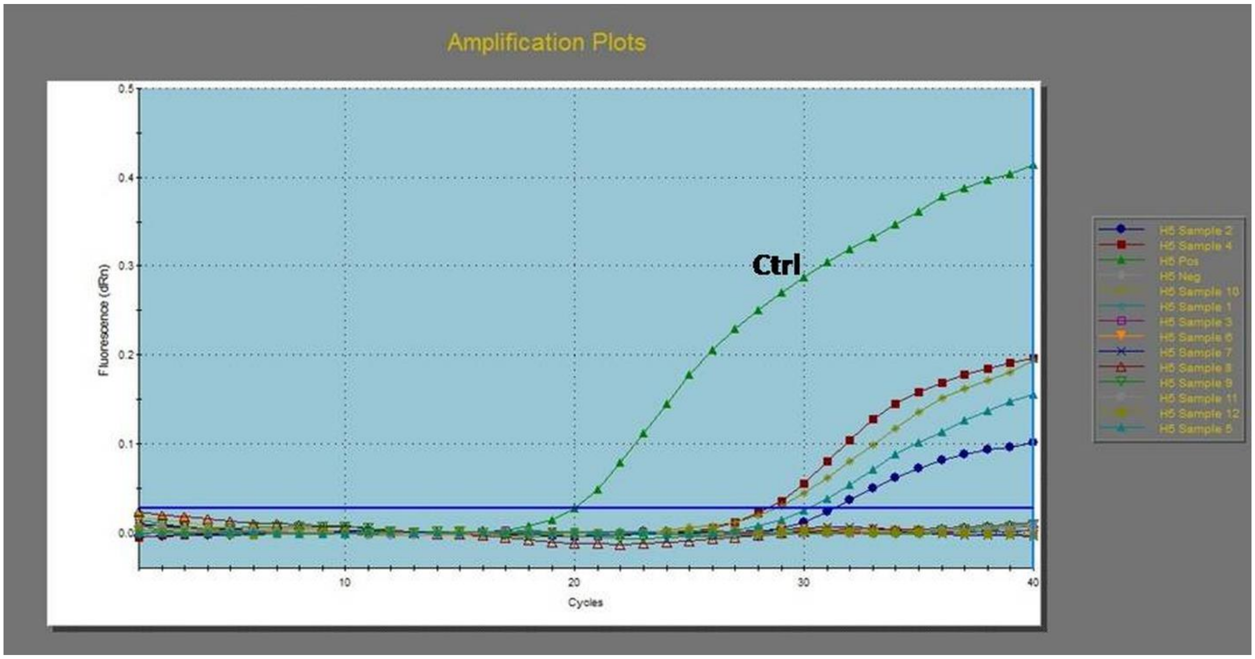

Supplement: Figure S1 [file peerj-06-5473-s003.pdf]
