# Supplementary material for: Molecular identification of avian influenza virus subtypes H5N1 and H9N2 in birds from farms and live bird markets and in respiratory patients
Source: PeerJ. 2018 Sep 5;6:e5473. doi: 10.7717/peerj.5473 (PMC6129142; doi:10.7717/peerj.5473)
Supplement: Table S1 [file peerj-06-5473-s004.docx]

**Table S1.** Sequences of the primers and probes used for avian influenza viruses.

| **Target** | **Primer/ probe sequence**  **5'-3'** | **Reference** |
| --- | --- | --- |
| **H5** | **H5LH1**  ACATATGACTAC CCACARTATTCA G | (Londt et al. 2008)^a^ |
|  | **H5RH1**  AGACCAGCT AYC ATGATTGC |  |
|  | **H5PRO**  [FAM]TCWACA GTGGCGAGT TCCCTAGCA[TAMRA] |  |
| **N1** | AIV-N1-F1  GGCATAATAACAGACACTATCAA | (Aguero et al. 2007)^b^ |
|  | AIV-N1-R1  CACATGCACATTCAGACTCT |  |
|  | AIV-N1-S1  [FAM]TCAGTATGTTGTTCCTCCA[TAMRA] |  |
| **H5 cleavage site** | **H5-kha-1**  CCT CCA GA**R** TAT GC**M** TA**Y** AAA ATT GTC | (Slomka et al. 2007)^c^ |
|  | **H5-kha-3**  TAC CAA CCG TCT ACC ATK CCY TG |  |
| **H9N2** | **H9F**  GGAAGAATTAATTATTATTGGTCGGTAC | (Ben Shabat et al. 2010)^d^ |
|  | **H9R**  GCCACCTTTTTCAGTCTGACATT |  |
|  | **H9 Probe**  [CY5]AACCAGGCCAGACATTGCGAGTAAGATCC[BHQ] |  |

^a^ Londt BZ, Nunez A, Banks J, Nili H, Johnson LK, and Alexander DJ. 2008. Pathogenesis of highly pathogenic avian influenza A/turkey/Turkey/1/2005 H5N1 in Pekin ducks (Anas platyrhynchos) infected experimentally. *Avian Pathology* 37:619-627. 10.1080/03079450802499126

^b^ Aguero M, Sanchez A, San Miguel E, Gomez-Tejedor C, and Jimenez-Clavero MA. 2007. A real-time TaqMan RT-PCR method for neuraminidase type 1 (N1) gene detection of H5N1 Eurasian strains of avian influenza virus. *Avian Diseases* 51:378-381. 10.1637/7642-050306r.1

^c^  Slomka MJ, Coward VJ, Banks J, Londt BZ, Brown IH, Voermans J, Koch G, Handberg KJ, Jorgensen PH, Cherbonnel-Pansart M, Jestin V, Cattoli G, Capua I, Ejdersund A, Thoren P, and Czifra G. 2007. Identification of sensitive and specific avian influenza polymerase chain reaction methods through blind ring trials organized in the European Union. *Avian Diseases* 51:227-234. 10.1637/7674-063006r1.1

^d^ Ben Shabat M, Meir R, Haddas R, Lapin E, Shkoda I, Raibstein I, Perk S, and Davidson I. 2010. Development of a real-time TaqMan RT-PCR assay for the detection of H9N2 avian influenza viruses. *Journal of Virological Methods* 168:72-77. 10.1016/j.jviromet.2010.04.019
