# Supplementary material for: Molecular identification of avian influenza virus subtypes H5N1 and H9N2 in birds from farms and live bird markets and in respiratory patients
Source: PeerJ. 2018 Sep 5;6:e5473. doi: 10.7717/peerj.5473 (PMC6129142; doi:10.7717/peerj.5473)
Supplement: Table S2 [file peerj-06-5473-s005.docx]

**Table S2.** Cycle threshold (Ct) values of the positive H5N1 and H9N2 positive samples from birds and humans.

| **Source** | **Subtype** | **Ct value** |
| --- | --- | --- |
| Duck-Farms | H5N1 | 11.2 |
| Duck-Farms | H5N1 | 12.1 |
| Duck-Farms | H5N1 | 13.2 |
| Duck-Farms | H5N1 | 16 |
| Pigeons-Farms | H5N1 | 12.1 |
| Pigeons-Farms | H5N1 | 15.1 |
| Pigeons-Farms | H5N1 | 19 |
| Pigeons-Farms | H5N1 | 12.8 |
| Pigeons-Farms | H9N2 | 32 |
| Pigeons-Farms | H9N2 | 31 |
| Duck-LBMs | H5N1 | 13.3 |
| Duck-LBMs | H5N1 | 14.2 |
| Duck-LBMs | H5N1 | 17.1 |
| Duck-LBMs | H5N1 | 28.8 |
| Duck-LBMs | H5N1 | 28.7 |
| Duck-LBMs | H5N1 | 12.5 |
| Duck-LBMs | H5N1 | 11.3 |
| Duck-LBMs | H5N1 | 12.1 |
| Duck-LBMs | H5N1 | 12.3 |
| Pigeons-LBMs | H5N1 | 35.2 |
| Pigeons-LBMs | H5N1 | 30 |
| Pigeons-LBMs | H5N1 | 31 |
| Pigeons-LBMs | H5N1 | 13.1 |
| Pigeons-LBMs | H5N1 | 12.4 |
| Pigeons-LBMs | H5N1 | 11.2 |
| Pigeons-LBMs | H5N1 | 14 |
| Quails-LBMs | H5N1 | 15.2 |
| Pigeons-LBMs | H9N2 | 31 |
| Pigeons-LBMs | H9N2 | 33 |
| Pigeons-LBMs | H9N2 | 31 |
| Pigeons-LBMs | H9N2 | 32 |
| Humans | H5N1 | 31 |
| Humans | H5N1 | 32.1 |
| Humans | H5N1 | 32.4 |
